# Application of a hybrid virtual-physical teaching model integrating mixed reality and 3D printing in clinical joint orthopedic education

**DOI:** 10.3389/fsurg.2025.1638619

**Published:** 2025-08-20

**Authors:** Honglin Wang, Aoshuang Xu, Wei Hua, Zhewei Ye, Lin Lu

**Affiliations:** ^1^ Department of Orthopedics, Renmin Hospital of Wuhan University, Wuhan, China; ^2^ Institute of Hematology, Union Hospital, Tongji Medical College, Huazhong University of Science and Technology, Wuhan, China; ^3^ Department of Orthopaedic Surgery, Union Hospital, Tongji Medical College, Huazhong University of Science and Technology, Wuhan, China

**Keywords:** mixed reality (MR), 3D-printed model, teaching mode, joint surgery education, virtual-physical integration, medical undergraduates

## Abstract

**Objective:**

The diagnosis and treatment of arthropathic orthopaedic conditions are inherently linked to anatomical structures, necessitating strong spatial visualization abilities in students. Providing intuitively accessible methods for students to master specialized knowledge presents a formidable challenge for educators. This study aims to evaluate the pedagogical value of integrating 3D-printed model with mixed reality (MR) technology in clinical orthopaedic surgery education.

**Methods:**

Thirty-six senior clinical medical undergraduates were randomized into two groups. The experimental group underwent training using the combined 3D- printed model and mixed reality (MR) technology, while the control group received traditional instruction. Learning outcomes were evaluated through standardized Objective Structured Clinical Examination (OSCE) assessments and questionnaires. Correlation analysis was conducted between total OSCE scores and questionnaire scores.

**Results:**

The experimental group achieved significantly higher OSCE scores compared to the control group (*p* < 0.05). Questionnaire analysis indicated that a majority of students in the experimental group perceived the integrated 3D printing and mixed reality (MR) teaching approach as innovative. They also reported significant improvements in anatomical comprehension, interest stimulation, teacher-student interaction, knowledge retention, learning efficiency, and practical skills (*p* < 0.05). Furthermore, OSCE total scores demonstrated strong positive correlations with all questionnaire subdomains (*r* > 0.8, *p* < 0.001). The strongest correlation was observed between learning efficiency and OSCE total scores (*r* = 0.918).

**Conclusion:**

Integrating 3D-printed model and MR technology into orthopedic joint surgery clinical teaching significantly boosts student performance, learning efficiency, and overall teaching quality, demonstrating strong potential for wider application.

## Introduction

Clinical teaching remains both a cornerstone and persistent challenge in medical education. High-quality, efficient clinical learning is decisive for accumulating clinical experience, enhancing clinical skills, and cultivating clinical thinking (1, 2). As a critical subspecialty of orthopedics, arthroplasty exhibits distinctive teaching characteristics due to its broad disease spectrum, technically complex procedures, and high surgical difficulty (3). However, traditional teaching methods fail to meet contemporary educational demands. On one hand, orthopedic clinical instruction predominantly relies on skeletal models and patient imaging as teaching aids (4). Yet skeletal models largely demonstrate only normal anatomy (5), while 3D images constrained by 2D display interfaces cannot authentically reconstruct the three-dimensional spatial conformation of the musculoskeletal system, thereby limiting students' spatial cognition and precise measurement of key clinical parameters (e.g., anteversion angle, abduction angle, teardrop sign, lower limb mechanical axis, flexion-extension gap, retroversion angle, prosthesis sizing and positioning), which ultimately constrains teaching effectiveness (6, 7). On the other hand, scarcity of educational resources, strained doctor-patient relationships, and limited hands-on opportunities create systemic challenges for medical students and junior surgeons in developing clinical reasoning and operative skills (8, 9). Consequently, actively exploring more effective instructional strategies has become imperative.

Teaching methods and tools are fundamental pathways to achieving educational objectives. With the rapid advancement of computer science and related disciplines, 3D printing and digital virtualization technologies have become widespread across various industries and are increasingly applied in the medical field (10). This offers significant opportunities to reform traditional medical teaching models and innovate clinical teaching practices. Specifically, 3D printing technology enables the creation of highly realistic physical models that replicate the features of actual cases (11). It concretizes abstract and complex anatomical structures, greatly enriches teaching resources, and allows trainees to engage with numerous case models before entering clinical practice (12). Simultaneously, Mixed Reality (MR) technology, as an advanced three-dimensional visualization tool, integrates both “augmented reality” and “augmented virtuality” functionalities (7, 13). It can stereoscopically display intricate multi-layered structures, seamlessly merge the real physical world with virtual information, and support multi-party real-time interaction (14, 15). Within the teaching context, MR technology converts patient 2D imaging data into intuitive 3D images and accurately superimposes/projects them onto real anatomical areas or operational spaces, achieving a “transparent visualization” effect (virtual anatomical structures are semi-transparently overlaid on physical models via MR to enable “perspective” observation of deep structures) that significantly deepens trainees' understanding of anatomical structures and surgical procedures while improving learning efficiency (15, 16). Critically, these two technologies demonstrate significant synergistic potential: 3D printed models provide a stable anatomical reference base and tactile feedback, while MR technology dynamically overlays simulated pathological variables, creating an immersive interactive experience. Together, they construct a comprehensive educational ecosystem that fuses physical reality with virtual information.

To fully integrate and leverage this synergistic “virtual-physical integration” technological advantage, this study innovatively proposes a “MR Immersive Classroom with 3D Printed Tactile Enhancement” teaching model. This model aims to systematically evaluate its practical efficacy in enhancing the effectiveness of orthopedic clinical teaching.

## Methods

### Design and participants

This study enrolled 36 trainee physicians undergoing clinical rotation in the joint surgery department between October 2023 and October 2024. Grouping was performed using a computer-generated random number table (SPSS 26.0 software), with an allocation concealment mechanism of sealed opaque envelopes. The control group received traditional teaching comprising physical examination instruction combined with multimedia lectures and imaging data interpretation. The experimental group received additional MR holographic immersive teaching and 3D-printed model-assisted instruction based on the control group's curriculum. Both the experimental group and the control group received training for 4 weeks, 3 times a week, 2 h each time. No statistically significant differences existed between the two groups regarding gender, age, rotation duration, or prior academic performance (*p* > 0.05). The MR device used was Microsoft HoloLens 2 (resolution 2K, field of view 96.1°), with the software platform being Windows Holographic.

### Inclusion and exclusion criteria

Inclusion Criteria: (1) Five-year clinical medicine program trainees; (2) First-time orthopedic rotation participants; (3) Completion of systematic anatomy foundation and clinical courses relevant to orthopedics; (4) Passing scores in all foundational and clinical stage assessments. Exclusion Criteria: (1) Declined participation; (2) Withdrew during the study; (3) Poor course compliance; (4) Severe communication barriers.

### Grouping method

All instructors recruited for this study held the academic rank of Associate Chief Physician or higher, possessed over 10 years of clinical experience, and were certified as qualified educators. All were proficient in independently performing joint replacement surgeries. Standardized instruction was delivered by the same instructor cohort across all teaching groups.

The control group strictly adhered to the *Surgery* curriculum framework. Focusing on four major disease categories—hip/knee osteoarthritis, femoral head necrosis, and femoral neck fractures—total hip arthroplasty and total knee arthroplasty were selected as core surgical procedures. Instructors developed standardized topic-specific PowerPoint presentations with annotated representative imaging cases, pre-recorded standardized lesson plans on pathological classifications and diagnostic-therapeutic principles. After obtaining informed consent, in-hospital patients were selected for clinical training, where instructors supervised students in medical history collection and bedside examinations. Finally, using medical simulators and synthetic bone models (Sawbones 3400 simulated models), instructors comprehensively analyzed articular anatomical features, surgical planning, and treatment strategies, while concurrently conducting skill training and assessment.

**Figure 1:**
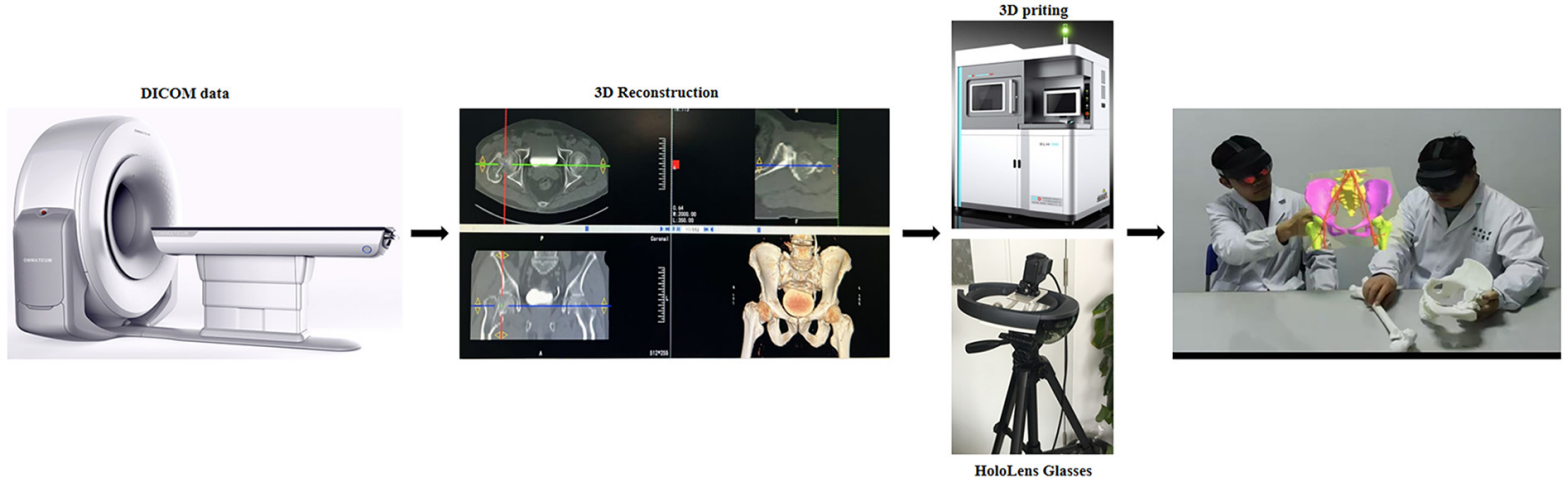
Experimental flow chart of this study.

The experimental group integrated digital technologies within the control group's pedagogical structure: Following systematic 3D/MR technology training, instructors selected representative hip/knee joint replacement cases. Preoperative and postoperative DICOM imaging data were imported into Mimics 20.0 software for 3D reconstruction. MR technology was then employed to generate interactive holographic virtual models (supporting multi-angle zoom observation), while synchronized 3D printing (1 mm layer thickness) produced 1:1 high-fidelity physical models. During instruction, the dual-modality approach (physical models + MR holograms) was synergistically implemented to dynamically demonstrate articular anatomy, surgical approaches, and therapeutic decision-making (Figure 1). Training concluded with skill practice and assessment on simulated teaching apparatuses.

### Teaching effectiveness evaluation

This study systematically evaluated the pedagogical efficacy of two instructional models through integrated objective assessments and subjective questionnaires. The objective evaluation employed standardized Objective Structured Clinical Examinations (OSCE) for both resident physician cohorts: a self-developed theoretical assessment scale (weighted 40%, maximum score 100) evaluated core knowledge domains including three-dimensional anatomical features of hip/knee joints, pathological classification of osteoarthritis, categorization of femoral neck fractures, and fundamental principles of joint replacement surgery. Simultaneously, a proprietary practical skills scale (weighted 60%, maximum score 100) comprehensively assessed six clinical competencies: clinical inquiry proficiency, standardized physical examination techniques, diagnostic reasoning logic, formal medical documentation, prosthesis sizing measurement with model selection, and anatomical decision-making for implant positioning.

Subjective evaluation was conducted via self-administered 5-point Likert scale questionnaires. Satisfaction metrics were quantified on a 100-point scale across three evaluative dimensions: teaching experience (instructional innovation, classroom interactivity, learning engagement), cognitive enhancement (knowledge comprehension depth, learning efficiency, knowledge mastery), and competency development (clinical practice capability advancement, professional learning motivation). These multidimensional metrics were ultimately synthesized into a comprehensive teaching satisfaction index.

### Statistical methods

Statistical analyses were performed using SPSS 26.0. Continuous data are presented as mean ± standard deviation (*x¯* ± *s*). Independent samples *t*-tests compared OSCE scores and questionnaire ratings between groups. Pearson correlation analysis examined relationships between total OSCE scores and questionnaire dimensions. Statistical significance was set at *p* < 0.05.

## Results

### OSCE assessment scores

The experimental group achieved significantly higher OSCE scores than the control group, with theoretical knowledge scores of 81.33 ± 3.12 points vs. 62.44 ± 4.51 points, comprehensive practical skills scores of 83.89 ± 2.97 points vs. 64.78 ± 5.67 points, and total OSCE scores of 82.87 ± 2.33 points vs. 63.84 ± 5.02 points, respectively. The analysis confirmed statistically significant differences between groups (*P* < 0.05), demonstrating superior performance in the experimental group (Table 1).

**Table 1:** Comparison of objective structured clinical examination (OSCE) scores between the two groups.

Group	Theoretical assessment scores	Practical assessment scores	Total scores
Experimental group (*n* = 18)	81.33 ± 3.12	83.89 ± 2.97	82.87 ± 2.33
Control group (*n* = 18)	62.44 ± 4.51	64.78 ± 5.67	63.84 ± 5.02
*t*	15.256	13.387	15.036
*p* value	<0.001	<0.001	<0.001

### Questionnaire survey

Analysis of questionnaire responses revealed significantly higher ratings in the experimental group across all eight dimensions compared to the control group (*p* < 0.001). Trainees in the experimental group widely endorsed the innovative teaching approach integrating 3D printing and MR technology, reporting substantial improvements in knowledge retention, learning efficiency, and practical skills. They further indicated enhanced anatomical comprehension, stimulated learning interest, and facilitated teacher-student interaction (*p* < 0.05), demonstrating robust effectiveness of the novel methodology (Table 2).

**Table 2:** Comparison of teaching satisfaction evaluation between the two groups.

Group	Teaching innovation	Comprehension ability	Classroom concentration	Learning efficiency	Instructional interactivity	Enhancement of clinical skills	Learning interest	Knowledge mastery
Experimental group (*n* = 18)	75.28 ± 2.49	83.33 ± 2.40	90.50 ± 3.24	88.28 ± 2.44	83.33 ± 2.40	81.28 ± 2.44	83.33 ± 2.40	81.33 ± 2.40
Control group (*n* = 18)	60.06 ± 2.69	72.94 ± 2.62	72.00 ± 2.63	67.00 ± 2.63	72.94 ± 2.62	67.00 ± 2.63	72.94 ± 2.62	68.06 ± 2.62
*t*	18.039	12.857	19.344	26.206	12.857	17.252	12.857	16.201
*p* value	<0.001	<0.001	<0.001	<0.001	<0.001	<0.001	<0.001	<0.001

### Correlation analysis between total OSCE scores and questionnaire ratings

A strong positive correlation was observed between total OSCE scores and all questionnaire dimensions (*r* > 0.8, *p* < 0.001). The correlations ranked in descending order of strength were: learning efficiency > course engagement > knowledge mastery > clinical practice skills improvement > comprehension ability = teaching interactivity = learning interest > teaching innovation. Notably, learning efficiency demonstrated the strongest correlation with total OSCE scores (*r* = 0.918, *p* < 0.001), highlighting its pivotal role in academic performance (Table 3).

**Table 3:** Pearson correlation coefficients between total OSCE scores and questionnaire scores.

Questionnaire scoring items	Correlation coefficient (*r*)	p value
Teaching innovation	0.826	<0.001
Comprehension ability	0.891	<0.001
Classroom concentration	0.904	<0.001
Learning efficiency	0.918	<0.001
Instructional interactivity	0.891	<0.001
Enhancement of clinical skills	0.895	<0.001
Learning interest	0.891	<0.001
Knowledge mastery	0.902	<0.001

## Discussion

Orthopedics, as a highly specialized clinical discipline with a complex knowledge system and constrained instructional hours, features content intrinsically linked to anatomy and imaging sciences (17). It demands rigorous comprehension of biomechanical principles and spatial cognition in three dimensions. Traditional didactic teaching proves inadequate for deep mastery of core knowledge—particularly in knee/hip arthroplasty education, where theoretical instruction must be synergistically reinforced with hands-on procedural training (18). Currently, most medical schools adhere to conventional pedagogy: didactic lectures using radiographic data (x-ray/CT) coupled with slide presentations, supplemented by limited small-group practical sessions. This model exhibits significant limitations: monotonous formats, inadequate spatial representation, low student engagement, and passive knowledge assimilation, collectively compromising educational effectiveness (19). To address these challenges, our study innovatively integrates 3D printing and MR technologies to establish a novel clinical teaching paradigm for joint surgery. By integrating virtual and physical elements, this approach overcomes persistent practical constraints and demonstrably enhances instructional efficacy.

Recent years have witnessed the deep integration of 3D printing, virtual reality (VR), and augmented reality (AR) technologies into medical education, driving systemic pedagogical transformation (13, 20). Within orthopedic training, while these technologies demonstrate significant potential, each exhibits inherent structural limitations when deployed independently. VR excels in providing exceptional 360° anatomical visualization and surgical planning within immersive environments, yet fails to support tactile-dependent procedures like implant placement due to the absence of haptic feedback (21). Conversely, AR enables interactive guidance through real-world overlays but lacks realistic force feedback mechanisms critical for simulating orthopedic interventions (7). Meanwhile, 3D printing addresses tactile needs via patient-specific 1:1 models that facilitate hands-on practice of plate/screw fixation, though its static nature restricts dynamic visual guidance for spatial navigation (22). Recognition of these complementary strengths has catalyzed integrated solutions, as demonstrated by Jade et al.'s AR-coupled 3D-printed ankle/foot model which requires further validation of anatomical accuracy and educational efficacy (23). This is further evidenced by the AEducaAR project confirming enhanced learning motivation, long-term knowledge retention, and 3D spatial comprehension through physical-AR fusion (24), alongside documented cases where patient-specific VR with 3D models significantly strengthened preoperative confidence (25). Collectively, hybrid approaches synergizing digital immersion with physical interactivity demonstrate superior pedagogical effectiveness over single-modality methods, establishing foundational groundwork for exploring deeply integrated paradigms like MR.

As an emerging holographic imaging modality, MR combines the strengths of AR and VR to enable real-time coexistence and interaction between physical and digital objects, demonstrating significant potential in medical instruction (7, 14, 15). MR-based holographic visualization renders complex 3D anatomical structures with exceptional fidelity, allowing less-experienced clinicians to intuitively comprehend disease-specific morphological characteristics and pathological evolution. Concurrently, its capacity for real-time interactivity and seamless virtual-physical integration establishes an innovative platform for high-fidelity procedural simulation (26). Our prior research has validated MR's substantive advantages in clinical applications including physician-patient communication, preoperative planning, intraoperative navigation, and remote surgical consultations (7). However, current MR technology remains constrained by reliance on purely visual simulation without tactile feedback—a critical limitation that particularly impedes skill acquisition for procedures requiring haptic perception. The advent of 3D printing addresses this sensory gap by enabling localized reality enhancement within virtual environments: patient-specific CT imaging data is utilized to fabricate photorealistic 1:1 physical models, thereby establishing a foundation for tactile interaction and simulated operative training (10).

This study integrates 3D-printed anatomical models with MR technology to advance clinical education in joint surgery. The 3D models establish a static anatomical reference framework, while the MR system introduces dynamic pathological variables, synergistically resolving the dual challenges of spatial cognition deficits and scarce practical resources inherent in traditional teaching methodologies. This integrated approach bidirectionally enhances students' visual-tactile perception. Results demonstrate that students trained with this 3D-MR paradigm significantly outperformed conventionally trained peers in both theoretical knowledge assessments (*P* < 0.05) and comprehensive practical evaluations, validating its efficacy as an instructional tool. This finding aligns with Li et al.'s research demonstrating that MR-integrated 3D models enhance trainees' mastery of complex surgical pathologies while significantly boosting learning engagement and self-directed initiative (27). Furthermore, this innovative virtual-physical integration surpasses traditional methods across pedagogical metrics including learning efficiency, instructional interactivity, knowledge mastery, and clinical competency development. Its operational practicality substantially stimulates learning motivation while strengthening concentration and comprehension.

Key advantages manifest through four interconnected dimensions: First, the fusion of MR holographic visualization and tangible 3D models transcends spatiotemporal limitations, enabling persistent 3D visualization of orthopedic pathologies. This immersive environment facilitates multidimensional understanding of disease characteristics, igniting curiosity that drives knowledge internalization (28). Second, for complex surgical concepts like fracture classification systems and associated procedures (e.g., arthroplasty or internal fixation), the technology provides comparative virtual-physical integration modeling and imaging analysis, enabling intuitive differentiation of pathological variations while accelerating knowledge acquisition. Third, MR's visual immersion coupled with 3D models' tactile feedback generates spatio-haptic convergence that elevates spatial reasoning and anatomical comprehension—particularly for critical parameters including femoral valgus angle, anteversion angle, teardrop sign, and prosthetic positioning. Fourth, direct manipulation of patient-specific 3D models enables synchronous knowledge application, where progressive analysis across 2D imaging, 3D visualization, and physical specimens cultivates diagnostic reasoning. This closed-loop training enhances clinical problem-solving capabilities through systematic integration of patient data, ultimately improving diagnostic accuracy and therapeutic planning. Notably, the strong positive correlation between OSCE scores and questionnaire results (*r* = 0.82, *P* < 0.001) confirms both instrument validity and enhanced learning experiences. The highest correlation between learning efficiency and OSCE performance suggests knowledge assimilation mediates efficacy gains.

Furthermore, this study has several limitations that warrant acknowledgment. First, the participant pool was confined to orthopedic trainees from a single institution with a relatively small sample size, potentially limiting the generalizability of findings. Single-center study (*n* = 36) has limited generalizability and multi-center validation (*n* > 200) is required in the future. Second, the non-blinded design coupled with post-randomization informed consent procedures may have introduced unavoidable selection bias. Third, implementing the integrated 3D printing and MR approach requires significantly greater preparation time and effort from instructors compared to conventional teaching methods. Finally, the primary reliance on examination scores as evaluation metrics lacks multidimensional assessment approaches, potentially failing to comprehensively reflect students' actual clinical competencies.

In conclusion, the virtual-physical integrated pedagogical paradigm—combining tangible 3D models with persistent MR holographic visualization—demonstrates distinct advantages in medical education. It effectively stimulates learner engagement and motivation, enhances instructional interactivity, and facilitates holistic understanding of representative orthopedic pathologies. This approach fosters a deeper comprehension of musculoskeletal disorders and merits further exploration and broader implementation.

## Data Availability

The original contributions presented in the study are included in the article/Supplementary Material, further inquiries can be directed to the corresponding author.
